# Case Report: Transcatheter aortic valve implantation using balloon-expandable bioprosthesis in patients with severe pure aortic regurgitation on noncalcified native valves: a series of cases

**DOI:** 10.3389/fcvm.2024.1365181

**Published:** 2024-04-26

**Authors:** Luciano de Moura Santos, Larissa Santos Luz, Vinicius Lelis Bastos, Tulio Assunção Barcelos, Frederico André Alves Abreu, Leonardo Cogo Beck, Mohammed Jamal Aldin Hilal Darnasser, Francisco de Assis Cruz, Luis Carlos Vieira Matos, Wenderval Borges Carvalho

**Affiliations:** ^1^ Department of Interventional Cardiology, Hospital Santa Lucia Sul, Brasilia, Brazil; ^2^ Department of Interventional Cardiology, Hospital Santa Lucia Norte, Brasilia, Brazil

**Keywords:** aortic regurgitation, transcatheter aortic valve implantation, pure native aortic valve regurgitation, balloon-expandable valve bioprosthesis, leak paravalvular

## Abstract

**Background:**

For individuals with pure aortic regurgitation (AR), transcatheter aortic valve implantation (TAVI) is cautiously recommended only for those with a high or prohibitive surgical risk. We aimed to describe the results of a case series of transcatheter implantation of a balloon-expandable aortic valve bioprosthesis (BEV) for the treatment of noncalcified native valve AR.

**Methods:**

From February 2022–November 2022, we performed TAVI in patients with severe pure AR. Cases were indicated on the basis of symptoms, high/prohibitive surgical risk, or patient refusal of conventional treatment.

**Results:**

Five patients underwent successful TAVI. The mean age was 81.9 ± 6.6 years, 3 (60%) female and 5 (100%) in NYHA class III or IV. The baseline echocardiogram showed an ejection fraction of 49.0 ± 10.6% and left ventricular end-systolic diameter 28.5 ± 4.7 mm/m². The average area of the aortic annulus was 529.1 ± 47.0mm² and the area oversizing index was 17.6 ± 1.2%. In the 30-day follow-up, there were no cases of prosthesis embolization, annulus rupture, stroke, acute myocardial infarction, acute renal failure, hemorrhagic complication or death. One patient required a permanent pacemaker and another had a minor vascular complication. The clinical follow-up were 19.8 months (16.7–21.8). During this period, all patients remained alive and in NYHA class I or II. One of the patients developed a moderate paravalvular leak.

**Conclusion:**

TAVI with a BEV proved to be safe and effective in this small case series of patients with noncalcified native valve AR in a follow-up longer than 1 year.

## Background

Surgical aortic valve replacement (SAVR) is the standard treatment for symptomatic patients or those with echocardiographic changes related to severe aortic regurgitation (AR) (1). However, only a third of patients indicated for SAVR actually undergo the procedure, with high/prohibitive surgical risk being the main cause (2). Transcatheter aortic valve replacement (TAVR) is already a well-established therapy for the treatment of aortic stenosis, including for patients at low surgical risk, particularly in consideration of age (3). According to ACC/AHA and ESC/EACTS guidelines, TAVI is not indicated for pure aortic regurgitation, except in carefully selected patients who are ineligible for SAVR (1, 3). In this way, disseminating the experience of this percutaneous treatment in different centers can help in understanding the implant technique as well as the clinical evolution of patients undergoing this treatment. We aimed to demonstrate the clinical and echocardiographic evolution over a 1-year follow-up in a small series of cases.

## Methods

This study was carried out in two hospitals in the same Brazilian city. From February 2022–November 2022, all consecutive patients diagnosed with pure and severe aortic regurgitation and limiting symptoms were evaluated by the Heart Team, composed of a clinical cardiologist, interventional cardiologist, cardiac surgeon, and anesthesiologist. TAVI was indicated by the Heart Team, even completely off-label in this situation, after confirmation of high/prohibitive surgical risk or in case of persistent refusal of surgical treatment by the patient. All patients were entered into a national TAVI registry (CAAE 36697620.0.1001.5485). The study protocol of Brazilian TAVI Registry was approved by the local ethics committee. All patients were informed about the off-label use of the prosthesis for aortic regurgitation treatment, and they agreed and signed the informed consent form before undergoing the procedure. Additionally, written informed consent was obtained from all patients for the publication of this case report. All underwent pre-procedure CT angiographic evaluation to decide on the type and size of the prosthesis as well as vascular access. Data from computed tomographic angiography was analyzed using the software OsiriX MD version 14.0 (Pixmeo SARL, Bernex, Switzerland) by a single operator and without industry participation, as it was an off-label procedure. All measurements were assessed in the systolic phase. The height of the left main and right coronary arteries was measured between the base of both and the aortic annulus. The sinus of Valsalva was measured at the largest diameter site. Aortic annulus was measured on a transverse double oblique plane perpendicular to the long axis of the ascending aorta. LVOT was measured 4 mm below the annulus. We calculated the ellipticity index of the aortic annulus by dividing the maximum diameter by the minimum diameter. The choice of the type of prosthesis was at the discretion of the operator. The procedure was performed in the catheterization laboratory under sedation or general anesthesia and using transthoracic or transesophageal echocardiography. Clinical endpoints were prospectively collected and included device success, procedural complications (in accordance with VARC-2 definitions), NYHA functional class, and survival of at least 1 year. Echocardiograms were performed during follow-up and compared with those performed immediately after the procedure. Statistical analysis was performed using MedCalc Statistical Software version 20.210 (MedCalc Software Ltd, Ostend, Belgium).

## Results

**Figure 1:**
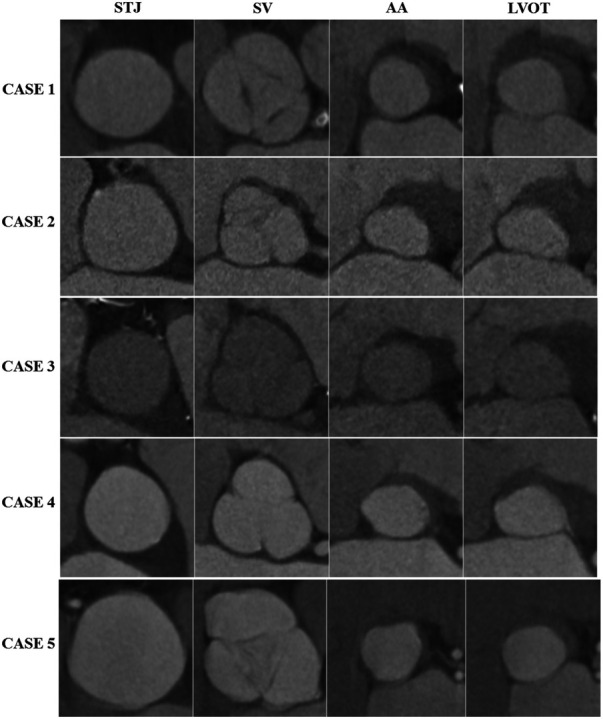
Demonstration of the shape and absence of calcifications in each component of the valve apparatus. AA, aortic annulus; LVOT, left ventricular outflow tract; STJ, sino-tubular junction; SV, sinos of vasalva.

Five patients, three of whom were female, underwent TAVI due to severe non-calcified AR, with a mean age of 81.9 ± 6.6. Four patients were contraindicated for SAVR by the Heart Team. One patient, despite having no contraindications for SAVR, refused surgery during six years of clinical follow-up. During this period, this patient developed moderate left ventricular dysfunction. After another refusal of surgery, TAVI was offered, and it was accepted by the patient. Three cases were performed electively, and two during hospitalization. The clinical and echocardiographic characteristics are presented in Table 1 and the tomographic and procedural characteristics in Table 2. The absence of calcification in all structures of the valve apparatus is shown in Figure 1. The procedure was successful in all cases, according to VARC II criteria. One procedure was performed under general anesthesia and 4 under sedation. In all cases, an Edwards Sapien 3 balloon-expandable aortic valve bioprosthesis (BEV) was deployed. The median oversizing was 17.10% (16.85–17.90). No patient underwent post-dilatation. All aortic annulus had an elliptical shape characterized by an index greater than 1.1. The areas of the aortic annulus and LVOT were respectively 529.1 ± 47.0 mm² and 569.3 ± 46.2 mm². The LVOT larger than the annulus was observed in four out of five patients.

**Table 1:** Clinical and echocardiographic characteristics of patients with pure non-calcified aortic regurgitation undergoing TAVI.

	Case 1	Case 2	Case 3	Case 4	Case 5	*Overall*
Age (years)	90	83	77	74	86	81.9 ± 6.6
Sex	Male	Female	Male	Female	Female	
BMI (kg/m^2^)	27.2	26.0	26.4	18.6	19.6	23.5 ± 4.1
BSA (m^2^)	1.83	1.56	1.79	1.46	1.36	1.60 ± 0.2
Clinical Follow-up (days)	660	654	595	536	393	567.6 ± 109.8
Echo follow-up (days)	529	585	595	536	252	465.4 ± 152.4
NYHA Class	III	III	IV	III	IV	
EuroSCORE II	6.01	5.77	8.61	4.64	8.22	6.65 ± 1.69
Hypertension	1	1	1	0	1	
Hyperlipidemia	0	0	0	0	0	
Current smoker	0	0	0	0	0	
Diabetes mellitus	0	0	0	0	0	
Previous PPM	0	0	1	0	0	
Previous stroke	1	0	0	0	0	
Previous MI	0	0	0	0	0	
Previous PCI	1	0	0	0	0	
Rhythm EKG	Sinus	AF	Pacemaker	Sinus	AF	
Pre-TAVI echocardiogram						
LVEDD (mm)	69	60	57	65	56	61.4 ± 5.5
LVESD (mm)	45	46	46	53	36	45.2 ± 6.0
LVESD (mm/m²)	24.6	29.5	25.7	36.3	26.5	28.5 ± 4.7
LVEF (%)	57	46	39	40	63	49 ± 10.6
Post-TAVI echocardiogram						
Maximum AVG (mmHg)	14	25	17	23	13.5	18.5 ± 5.2
Mean AVG (mmHg)	8	16	10	14	8.7	11.3 ± 3.4
EOA	1.45	1.7	2.16	1.48	2.06	1.77 ± 0.3
LVESD (mm/m²)	17.5	22.4	22.3	27.4	22.1	22.3 ± 3.5
LVEF (%)	62	61	42.6	59	65.8	58.1 ± 9.0

Values are presented as *n* or mean ± standard deviation.

AF, atrial fibrillation; AVG, aortic valve gradient; BMI, body mass index; BSA, body surface area; Echo, echocardiographic; EKG, electrocardiogram; EOA, effective orifice area; LVEDD, left ventricular end-diastolic diameter; LVEF, left ventricular ejection fraction; LVESD, left ventricular end-systolic diameter; MI, myocardial infarction; NYHA, New York heart association; PCI, percutaneous coronary intervention; PPM, permanent pacemaker; TAVI, transcatheter aortic valve implantation. 1 = yes, 0 = no.

**Table 2:** Computed tomography characteristics of patients with pure non-calcified aortic regurgitation undergoing TAVI.

	Case 1	Case 2	Case 3	Case 4	Case 5	*Overall*
Area of the aortic annulus, mm²	533.6	477.4	603.3	527.8	503.8	529.1 ± 47.0
Perimeter of the aortic annulus, mm	82.4	80.6	88.3	83.7	81.4	83.3 ± 3.0
Maximum annulus diameter, mm	27.6	28.7	30.1	30.9	28.5	29.1 ± 1.8
Minimum annulus diameter, mm	24.4	21.3	25.1	23.4	24.7	23.8 ± 1.5
Average annulus diameter, mm	26.1	24.7	27.7	25.9	25.3	25.9 ± 1.1
Ellipticity index	1.13	1.35	1.20	1.32	1.15	1.23 ± 0.1
Left ventricular outflow tract, mm²	561.7	596.5	626.8	558.3	503.4	569.3 ± 46.2
Height of the LMCA, mm	15	4.2	11.5	10.8	10	10.3 ± 3.9
Height of the RCA, mm	23.7	10.5	16.1	14	18	16.5 ± 4.9
Left coronary sinus, mm	46.3	37.9	38.2	41.3	48.3	42.4 ± 4.7
Rigth coronary sinus, mm	43.9	35.6	40	41.3	45.8	41.3 ± 3.9
Non-coronary sinus, mm	44	33.6	39.5	38.7	43	39.7 ± 4.0
STJ diameter, mm	39	38	37	35.6	49	39.7 ± 5.3
Transcatheter Heart Valve	Sapien 3	Sapien 3	Sapien 3	Sapien 3	Sapien 3	
Size	29	26	29	29	29	
Area oversizing index, %	17.1	17.3	19.7	16.9	16.7	17.6 ± 1.2

Values are presented as *n* or mean ± standard deviation.

LMCA, left main coronary artery; RCA, right coronary artery; STJ, Sino-tubular junction.

The technique used for valve implantation (Supplementary Video S1) did not differ significantly from the usual one used for cases of aortic stenosis, except for avoiding high implants to minimize the possibility of embolization and using, in some cases, two Pigtail catheters to better identify the aortic annulus and provide more oversizing. In summary, after initially positioning the prosthesis, we performed an angiogram to confirm the central marker at the level of the aortic annulus. We then turned on the pacemaker at 180 bpm, waited a few seconds to drop the pressure significantly, and partially insufflated the prosthesis. At this point, a new angiogram was performed to confirm the position before complete insufflation. We waited for a total of 5 s during complete insufflation and then initiated deflation. Afterward, we turned off the pacemaker. Following this, we checked the performance of the prosthesis and assessed potential complications using echocardiography. We also kept the guidewire inside the ventricle for longer than usual in order to prevent the prosthesis from rotating within the ventricle, which could impede systole in case of embolization into the ventricle. All patients showed significant improvement in aortic regurgitation, with no residual leak in 1 patient, minimal in 3 patients and mild in 1 patient. Supplementary Video S2 demonstrates, according to echocardiographic evaluation, the improvement in aortic regurgitation after TAVI in one of the patients. A permanent pacemaker was implanted in one of the patients the day after the procedure, at the discretion of the attending physician due to pre-existing severe sinus bradycardia. Another patient experienced occlusion of the external iliac artery after the removal of the 14F introducer, and angioplasty with a stent was successfully performed without complications. There were no cases of annulus rupture, prosthesis embolization, acute myocardial infarction, stroke, hemorrhagic or major vascular complications.

The clinical and echocardiographic follow-up times were, respectively, 19.8 months (16.7–21.8) and 17.9 months (11.1–19.5). At least 1-year follow-up, all-cause mortality was 0%, and all patients showed improvement in NYHA functional class (from NYHA III or IV pre-procedure to I or II post-procedure and during clinical follow-up). A significant reduction in the indexed left ventricular end-systolic diameter (LVESD) and left ventricular ejection fraction (LVEF) was observed (28.5 ± 4.7mm/m² × 22.3 ± 3.5mm/m², *p* = 0.003 and 49.0 ± 10.6% × 58.1 ± 9.0%, *p* = 0.05, respectively). Regarding paravalvular leak, four patients maintained the same result during follow-up. One patient developed a moderate leak two months after implantation, which remained stable after 1.5 years of follow-up. Despite being asymptomatic and without hemolysis, no intervention was considered necessary. Notably, this patient also demonstrated an improvement in LVESD (36.3 mm/m²–27.4 mm/m²) and LVEF (40%–59%).

## Discussion

In this small series of TAVI cases with balloon-expandable aortic valve bioprosthesis in patients with pure non-calcified AR, we observed: (1) a feasible and safe procedure in all cases, (2) symptom improvement, and (3) few complications associated with the procedure.

The use of transcatheter heart valve (THV) for the treatment of severe AR is not widespread, despite the large number of patients who do not have minimal clinical conditions to undergo SAVR. The main reason is that the vast majority of THV used for the treatment of aortic stenosis were designed to anchor in the calcified structures of the valve apparatus. The absence of calcium or its minimal presence in cases of pure AR greatly complicates the procedure, adding risks such as embolization or migration of the prosthesis (4). Therefore, the use of THV in a non-calcified aortic valve such as pure AR constitutes a completely off-label use, which is a hindering factor for conducting randomized trials. So, the existing experience in this scenario is mainly derived from isolated cases, case series, or meta-analyses. Additionally, there may be underreporting of unsuccessful cases in attempting to treat pure aortic regurgitation with the use of current THV.

However, despite the predominant use of non-dedicated devices in most reports in the literature, there has been a significant improvement in the outcomes of TAVI in AR with the development of new-generation devices compared to the previous generation (5). This improvement is observed irrespective of whether balloon-expandable or self-expandable valves are utilized (6–8).

In our cases, we performed implants with oversizing ranging from 16.7%–19.7%. Regarding the implant technique for balloon-expandable valves, the most significant modification concerned oversizing. In the literature, oversizing ranges between 15% and 35% were reported (9). The oversizing used was less than that verified in many case reports or meta-analyses. However, we must emphasize that TAVR in pure AR is an off-label procedure, and there is not a specific rule on how to do the deployment. We used an oversizing greater than usual in on-label cases of TAVR for aortic stenosis but avoided extreme oversizing due to the fear of annulus rupture. This complication, in many situations, can be more difficult to manage and more fatal than embolization of the prosthesis. Thus, it becomes imperative to carefully weigh and counterbalance these risks.

In our series, we used only BEV. Despite our group having experience with both BEV and self-expanding valve (SEV), our first case involved a patient with a huge aorta angulation (73 degrees) and a large annulus. These characteristics, combined with a complete absence of calcium, caused us greater concern about the possibility of embolization with the use of a SEV. After the successful outcome of this initial case, we opted to use BEV in subsequent cases. However, according to the literature, SEV has the same probability of success as BEV (once again, both off-label). It is known that BEV, especially the Edwards Sapien 3, expand the outflow and inflow more than the mid valve when overexpanded. As mentioned, four out of five patients presented an LVOT larger than the annulus. During the implant, the oversizing aimed to achieve not only greater fixation on the annulus due to higher pressure exerted than usual but also overexpansion in the outflow and inflow to minimize the possibility of embolization. Perhaps the difference in area between the annulus and the LVOT contributed to achieving this. But we would need to have performed computed tomographic angiography (CTA) after the implants to confirm if this indeed occurred.

According to ESC and ACC guidelines, one of the criteria supporting the indication for SAVR is an LVESD greater than 50 mm. Only one of the five patients had an LVESD > 50 mm (mean of 45.2 mm). However, these patients had a small body surface area (1.60 ± 0.2m²), and the average indexed LVESD was 28.5 mm/m^2^, in line with the indexed value recommended by ESC and ACC (>25 mm/m^2^). Nevertheless, the indication for SAVR in symptomatic patients or those with reduced left ventricular ejection fraction is independent of the LVESD value.

**Figure 2:**
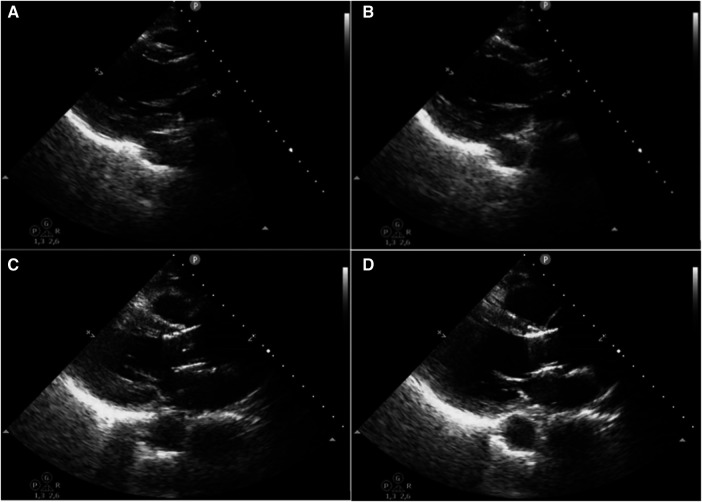
Comparison of the prosthesis positioning immediately after implantation with its position two months later. A = systole immediately after implantation; B = diastole immediately after implantation; C = systole two months later; D = diastole two months later.

One potential etiology for the moderate leak observed during the follow-up of case number 4 is the late migration of the prosthesis downwards, from its initial position. Although no CTA was performed, this suspicion arose from the comparison between the transesophageal echocardiogram performed at the time of implantation and the one conducted two months later when the leak was observed, as shown in Figure 2. It is important to mention that no paravalvular leak was detected in an echocardiogram performed one month after implantation.

In contrast to aortic stenosis, where the etiology is degenerative calcification, chronic AR has diverse causes, including leaflet anomalies, distortion, or dilation of the aortic root secondary to rheumatological diseases, medication use, and infectious diseases. This variability in etiology opens the door to different post-implantation outcomes based on the underlying cause (1).

As already demonstrated, we also observed improvements in both the patients' clinical condition and parameters related to the echocardiogram. This demonstrates the potential of TAVI in patients who are not eligible for SAVR, based on this small series of cases.

## Conclusions

The current series of cases demonstrated the technical feasibility of the procedure and favorable clinical outcomes over a follow-up period exceeding one year in patients with noncalcified native aortic regurgitation who underwent TAVI. However, the emergence of a case of paravalvular leak in the follow-up, possibly due to migration, raises awareness about the behavior of the prosthesis in native, non-calcified valves.

## Data Availability

The original contributions presented in the study are included in the article/Supplementary Material, further inquiries can be directed to the corresponding author.
